# The Efficacy of an Energy-Restricted Anti-Inflammatory Diet for the Management of Obesity in Younger Adults

**DOI:** 10.3390/nu12113583

**Published:** 2020-11-22

**Authors:** Gordana Kenđel Jovanović, Ines Mrakovcic-Sutic, Sandra Pavičić Žeželj, Branislav Šuša, Dario Rahelić, Sanja Klobučar Majanović

**Affiliations:** 1 Department of Health Ecology, Teaching Institute of Public Health of Primorsko-goranska County, Krešimirova 52a, 51000 Rijeka, Croatia; sandrapz@medri.uniri.hr; 2 Department of Physiology, Immunology and Pathophysiology, Faculty of Medicine, University of Rijeka, Braće Branchetta 20/1, 51000 Rijeka, Croatia; ines.mrakovcic.sutic@medri.uniri.hr; 3 Department of Health Ecology, Faculty of Medicine, University of Rijeka, Braće Branchetta 20/1, 51000 Rijeka, Croatia; 4 General Hospital Pula, Santoriova 24a, 52100 Pula, Croatia; drbranislav.susa@gmail.com; 5 Vuk Vrhovac University Clinic for Diabetes, Endocrinology and Metabolic Diseases, Merkur University Hospital, Dugi dol 4a, 10000 Zagreb, Croatia; dario.rahelic@gmail.com; 6 School of Medicine, University of Zagreb, Šalata ul. 2, 10000 Zagreb, Croatia; 7 School of Medicine, Josip Juraj Strossmayer University of Osijek, 31000 Osijek, Croatia; 8 Department of Endocrinology, Diabetes and Metabolic Diseases, Clinical Hospital Centre Rijeka, Krešimirova 42, 51000 Rijeka, Croatia; sanja.klobucar@medri.uniri.hr; 9 Department of Internal Medicine, Faculty of Medicine, University of Rijeka, Braće Branchetta 20/1, 51000 Rijeka, Croatia

**Keywords:** anti-inflammatory, cytokines, diet, inflammation, obesity, weight reduction programs

## Abstract

There is growing evidence of the dietary impact on obesity-induced low-grade chronic inflammation and the associated chronic non-communicable diseases modification. We determined changes in body composition and cardiometabolic and inflammatory status of participants with obesity after 24 weeks of a dietary intervention based on an energy-reduced anti-inflammatory diet and examined the relationship of these changes with changes in the inflammatory potential of the diet. The anthropometric and body composition parameters of 81 participants (average age of 43 years, 74 women) were assessed. Metabolic status was determined using the glycemic and lipid statuses, and the cardiometabolic index and inflammatory status were determined using the concentration of high-sensitivity C-reactive protein (hs-CRP), interleukin 6 (IL-6), and tumor necrosis factor α (TNF-α). The inflammatory potential of the diet was assessed using the Dietary Inflammatory Index (DII^®^). Intervention with an anti-inflammatory diet resulted in a significant reduction in body weight and visceral adipose tissue and caused improvements in the participants’ cardiometabolic and inflammatory statuses. The anti-inflammatory diet was shown to be effective regarding obesity management. The study data could advance current scientific knowledge in the field of inflammation and diet, provide guidelines for obesity management, and find its application in routine clinical practice.

## 1. Introduction

Obesity is a key modifiable risk factor that increases the burden of non-communicable diseases (NCDs), such as type 2 diabetes (T2D), cardiovascular disease (CVD), and cancer, which makes it one of the greatest clinical and public health challenges of the 21st century [1]. The current obesity prevalence in most European countries is around 20% [2], without a systematic difference concerning gender [3]. The association between obesity and NCDs relies on the theory of chronic, low-grade inflammation. Serum levels of inflammatory markers, such as C-reactive protein (CRP), tumor necrosis factor α (TNF-α), and interleukin 6 (IL-6), correlate with body mass index across the broad range of obesity [4]. The effects of the inflammatory markers that are triggered by excessive adipose tissue have been reported on insulin signaling pathways, resulting in insulin resistance, endothelial dysfunction, and further activation of the innate immune system, eventually progressing to T2D and other cardiometabolic disorders [4,5,6,7]. A reduction in excessive body fat mass, particularly visceral fat, has been shown to decrease obesity-induced inflammation, thus leading to the amelioration of obesity-related comorbidities [8,9]. 

There is a growing body of evidence linking diet with the immune system functioning and the modulation of the inflammatory response [10,11]. An unhealthy diet that is high in saturated fats and refined sugars seems to be one of the main factors implicated in the development of obesity and other NCDs [10], while a diet rich in fruit, vegetables, extra virgin olive oil, legumes, whole grains, fish, nuts, cocoa, coffee, tea, and red wine, similar to the Mediterranean diet, has demonstrated beneficial effects on cardiometabolic risk reduction [12]. The abovementioned food items contain various bioactive components that exhibit anti-inflammatory activity [12,13], and if consumed as a dietary pattern, it could be considered an anti-inflammatory diet, although currently, there is still no single, comprehensive, universally accepted definition of such a diet. Of all dietary patterns, the Mediterranean diet has been shown to have the highest anti-inflammatory potential [14], with beneficial effects on weight regulation, inflammation, and obesity-related cardiometabolic derangements [15,16,17,18,19,20]. A recently published umbrella review of meta-analyses of randomized controlled trials on the impact of popular dietary patterns on anthropometric and cardiometabolic parameters concluded that among all the diets evaluated, the Mediterranean diet had the strongest and most consistent evidence of an improvement in weight, body mass index (BMI), and cardiometabolic risk factors [21]. 

## 2. Materials and Methods 

The concept of an energy-restricted anti-inflammatory diet for obesity management among younger adults was protocoled as a randomized clinical trial to evaluate its applicability and efficacy [22]. The study was registered with clinicaltrials.gov: NCT03987776. Herein, we report its preliminary results.

### 2.1. Subjects

The participants were recruited during their first visit to the obesity outpatient clinic at the Clinical Hospital Centre Rijeka, Croatia. The inclusion criteria were as follows: participants of both genders, ages 18–50 years, BMI ≥ 30 kg/m^2^ with or without obesity-related complications, and stable body weight for the previous three months. 

Exclusion criteria were cigarette smoking within 6 months before the study’s initiation; chronic heart, kidney, and/or severe liver disease; malignant disease or history of malignant disease; use of anti-inflammatory or immunosuppressive drugs or medications for weight loss; changes in chronic medications; active infection or surgical procedure in the previous three months; food allergy or intolerance to any anti-inflammatory diet constituent; pregnancy and/or lactation.

The presence of metabolic syndrome was assessed according to the definition from the International Diabetes Federation Task Force on Epidemiology and Prevention [23]. 

### 2.2. Study Design

This six-month, two-arm randomized controlled trial with a two (group) by two (time) repeated measures design was conducted between March and October 2019 at Clinical Hospital Centre Rijeka, Croatia. Following the study presentation and baseline assessments, the recruited patients were randomly allocated to the anti-inflammatory diet (AID) group or the control diet (CD) group using a web-based randomization system (https://www.random.org/), which was run by trained medical personnel who were not engaged in any other study procedure. The study was approved by the ethics committee of the Clinical Hospital Centre Rijeka (Reg. No: 2170-29-02/15-16-4, 31 January 2017) and was conducted in line with the Declaration of Helsinki. Written informed consent was obtained from all participants before they participated in the study.

### 2.3. Intervention

All study participants attended monthly educational workshops that were managed by a clinical dietitian. The AID group participants were strongly encouraged to follow an energy-restricted diet, based on low glycemic foods, whole-grain products, legumes, colorful vegetables and fruits, nuts, seeds, marine fish, olive oil, green/black tea, and multiple spices and herbs. The CD group participants were strongly encouraged to follow an isocaloric standard diet protocol for bodyweight reduction (55–60% carbohydrates, 25–30% fat, 15–20% protein) [24]. A detailed description of the dietary interventions was provided in a study protocol [22]. An overlap in the recommended daily intake of vegetables, fruits, legumes, whole grains, nuts, green tea, and herbs between the AID and CD groups was bridged with more frequent use of olive oil, colorful low glycemic index vegetables and fruits, nuts, seeds, onion, garlic, various spices, marine fish, fermented dairy products, and with the avoidance of red and processed meat and industrially processed foods in AID group. 

The daily resting energy expenditure was calculated for each participant according to Mifflin–St. Jeor’s equation [25] using their baseline anthropometric measurements, which was then multiplied by the activity factor based on information from the physical activity questionnaire. The value obtained from these equations was reduced by 25%, thus providing the recommended energy intake for each participant. The adjustments of the number and quantity of servings of each food group were made accordingly.

During the workshops, meal planning with recipes, food serving sizes, the consumption of specific foods, and personal goal-setting were discussed. Participants who had missed the educational workshop were provided with workshop materials. If diagnosed with vitamin D deficiency according to the baseline serum vitamin D status, the participants were recommended to take vitamin D supplements. The intake of other dietary supplements during the trial was noted and included in all dietary calculations.

To assess the compliance with given dietary recommendations, participants were asked to fulfill a 3-day food intake record (covering two weekdays and one weekend day) before a monthly group meeting (overall six 3-day food intake records). After being evaluated, the dietary records were discussed with each participant. Participants whose adherence to a dietary intervention was less than 75% were considered as non-compliant and were discontinued from the trial.

### 2.4. Anthropometry and Body Composition

The anthropometric measurements were taken using a calibrated integrated weighing platform (seca mBCA 515, secaGmbH&Co. KG, Hamburg, Germany), with an accuracy of ±50 g up to 100 kg and a digital stationary stadiometer with the wireless transmission (seca 264, secaGmbH&Co. KG, Hamburg, Germany), with an accuracy of ±2 mm. The BMI was calculated as the weight (in kilograms) divided by the square of the height (in meters). Waist circumference (WC) was measured midway between the lowest rib and the upper border of the iliac crest in the medial axillary line at the end of a normal exhalation using a non-stretchable measuring tape (seca 201, secaGmbH&Co. KG, Hamburg, Germany). The body composition was measured via the bioelectrical impedance method using the measuring platform (seca mBCA 515, secaGmbH&Co. KG, Hamburg, Germany) with four pairs of electrodes, where one was placed on each arm and foot during the measurement. The impedance was measured with a current of 100 μA at frequencies between 1 and 1000 kHz. The duration of each bioelectrical impedance measurement was 75 s. Participants were measured in a standing position with hands outstretched. All participants were advised not to exercise for 12 h and not to drink alcohol for 24 h before measuring the impedance. 

### 2.5. Cardiometabolic Parameters

The cardiometabolic parameters included fasting plasma glucose, HbA1c, insulin, total cholesterol, low-density lipoprotein cholesterol (LDL-C), high-density lipoprotein cholesterol (HDL-C), and triglycerides, which were measured at the baseline and the end of the trial. Whole venous blood samples of each participant were collected in the morning by experienced medical personnel. All participants were instructed to fast for at least 12 h and to restrain from physical activity and alcohol for 24 h before the blood sampling. Fasting plasma glucose, HbA1c, and blood lipids were analyzed using photometry on an Olympus 5800 (Olympus, Center Valley, PA, USA). The chemiluminescent immunoassay method was used to determine the vitamin D (Cobas E601, Roche, Mannheim, Germany) and insulin concentrations (Immulite 2000xp, Siemens, Eschborn, Germany).

### 2.6. Inflammatory Parameters

To evaluate the inflammatory status, the concentrations of high-sensitivity C-reactive protein (hs-CRP), IL-6, and TNF-α were determined. hs-CRP was assessed using the immunoturbidimetry method on an Olympus 5800 (Olympus, Center Valley, PA, USA), while ELISA assay kits were purchased from eBioscience™ (Thermo Fisher Scientific, Waltham, MA, USA) and used for the measurement of IL-6 and TNF-α. Standard curves were constructed for the determination of each cytokine concentration according to the manufacturers’ instructions. 

### 2.7. The Inflammatory Potential of the Diet

The inflammatory potential of each participant’s diet was assessed with the dietary inflammatory index (DII^®^) [26], which is based on the data from the food frequency questionnaire (FFQ), which has already been applied to the Croatian population [27], together with the data obtained from the 3-day food intake diaries recorded each month of the study, as previously described. The FFQ was recorded at baseline and at the end of the trial to obtain the frequency (from once per month to a few times per day) and portion size (small, medium, and large) information about food and beverage consumption. In addition to the standard list of 97 food items, for this trial, an additional 36 food items and 14 herbs and spices with anti-inflammatory properties were included in the FFQ. 

To calculate the energy and dietary components intake, the Croatian food composition database [28] was used. The intake of certain nutrients, such as caffeine, β-carotene, omega-3, and omega-6 fatty acids, was estimated using the Danish [29] and American food composition database [30], the Phenol-Explorer 3.0 database [31], and the United States Department of Agriculture (USDA) [32]. As thermal processing, including domestic cooking, has long been known to influence the contents of the various polyphenols present in certain food [33], the total polyphenols were multiplied by their retention factors. Polyphenols losses due to boiling were calculated with a retention factor of 0.59, with 0.56 for frying, and 0.50 for baking [34]. 

For the DII^®^ calculation, the provided energy and dietary data from the FFQ or 3-day food diary of each participant were first linked to the global means and standard deviations of the food and nutrients intakes from 11 nations to calculate the z-scores [26], then converted to a percentile and centered to minimize the “right skew” by doubling the value and subtracting 1. The provided percentile score of each food parameter was then multiplied by the respective inflammatory effect score to provide the food parameter-specific DII^®^ score [26]. The overall DII^®^ score for each participant was the summation of forty-five food parameter-specific DII^®^ scores [26]. The positive values of the DII^®^ score indicated a pro-inflammatory diet, and negative values indicated an anti-inflammatory diet [26].

### 2.8. The Outcomes

To examine the efficacy of an energy-restricted anti-inflammatory diet in the management of obesity, the changes in body weight, body composition, cardiometabolic (fasting blood glucose, HbA1c, insulin resistance, lipid profile, cardiometabolic index) and inflammatory (hs-CRP, IL-6, TNF-α) parameters following a 6-month dietary intervention were assessed. 

Insulin resistance was calculated using the Homeostatic Model Assessment for Insulin Resistance (HOMA-IR) [35], according to the formula: HOMA-IR = (insulin (mU/L) × glucose (mmol/L))/22.5. The cardiometabolic index (CMI) was used to assess the presence of diabetes risk and the progression of atherosclerosis as an indicator of visceral adipose tissue distribution and dysfunction according to the formula: CMI = (triglycerides (mmol/L)/high-density lipoproteins (mmol/L)) × (waist circumference (cm)/body height (cm)) [36]. All changes were calculated using z-scores ((mean after intervention – baseline mean)/baseline mean) ×100.

### 2.9. Statistical Analysis

For the purpose of this pilot study, the required sample size was calculated using MedCalc (MedCalc for Windows, version 15.0, MedCalc Software, Ostend, Belgium) based on the changes in hs-CRP levels observed in participants included in an energy-restricted Mediterranean-inspired low glycemic load AI dietary intervention [37]. To detect a meaningful difference in the level of hs-CRP, with an α level of 0.05, power equal to 0.8, group size ratio 1:1, and using the *t*-test for repeated measures, it was calculated that 33 participants per group were required. The independent *t*-test, Mann–Whitney *U* test, and χ^2^test were used to compare the study groups’ characteristics at baseline and week 24. To test the differences in all participants’ characteristics obtained within the study, the *t*-test and Wilcoxon test, ANOVA, and Friedman’s ANOVA test for repeated measurements were used. Linear regression analysis was used to evaluate the potential relationships and correlations between the inflammatory potential of the diet as an independent variable with anthropometric, metabolic, and inflammatory variables as dependent variables. Adjustments were made for age, sex, educational level, physical activity, and obesity degree. Models were not adjusted for the total energy intake because it is one of the DII^®^ components. All tests were run with Statistica 12.7 for Windows (Statsoft Inc, Tulsa, OK, USA), which were regarded as two-tailed, and *p*-values < 0.05 were considered to be statistically significant.

## 3. Results

### 3.1. Study Participants

**Figure 1:**
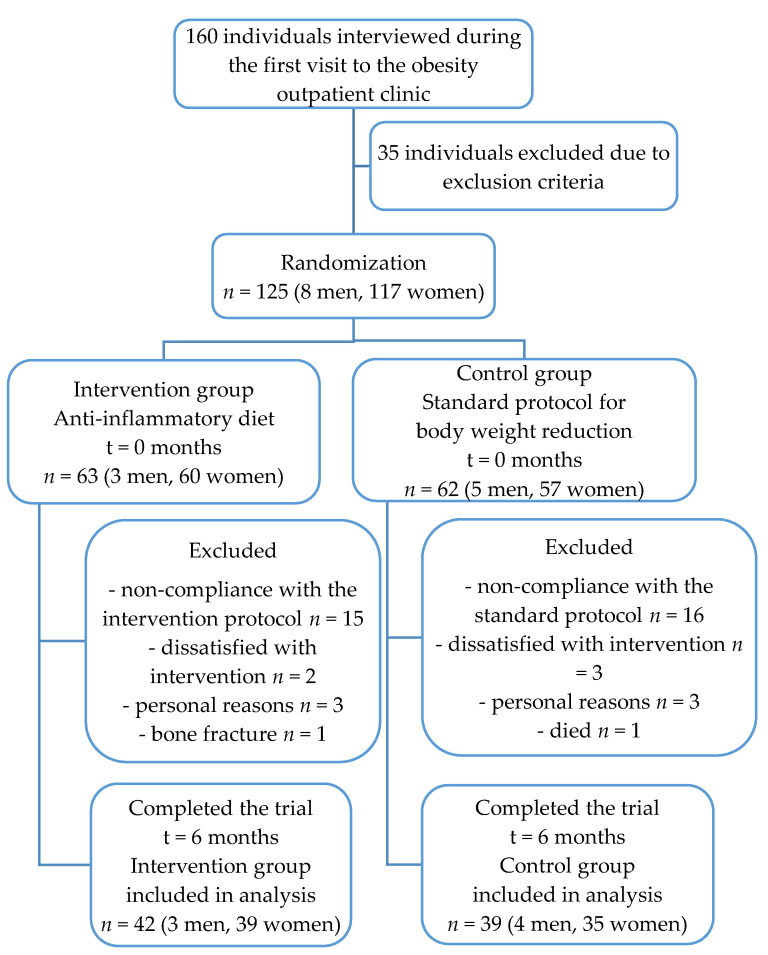
The flow diagram of participants included in the study.

The flow of participants throughout the study is shown in Figure 1. Out of 125 participants fulfilling the inclusion and exclusion criteria, 63 were randomized to the AID group and 62 into the CD group. Overall, 44 participants were excluded from the study mainly due to non-compliance with the dietary recommendations. Therefore, 81 participants (42 (51.9%) in the AID group and 39 (48.1%) in the CD group) completed this 6-month study.

Concerning gender, there was no statistically significant difference between the studied groups (*p* = 0.619) but there were significantly more women (92.9% and 89.7%) than men (7.1% and 10.3%) in both the AID and CD groups, respectively (*p* < 0.001) (Table 1). The mean age of the AID group participants was 44 years, and 42 years in the CD group, without a statistically significant difference (*p* = 0.178). There were significantly more married participants (90.5% and 84.5%) in both groupscompared with other relationship statuses. In the AID group, significantly more participants had a university education (61.9% in AID vs. 56.4% in CD, *p* = 0.002) compared with other education levels, and significantly more participants were employed (95.2%, *p* = 0.033) than unemployed in the AID group, while in the CD group, almost a quarter of participants were unemployed (23.1%). Most of the participants in both groups were physically inactive and hadfirst-degree obesity. Almost half of the participants in the AID group met the International Diabetes Federation-criteria for metabolic syndrome along with one-third in the CD group. Additionally, significantly more participants in the AID group were diagnosed with autoimmune thyroid disease (23.8% in AID vs. 7.7% in CD, *p* = 0.048).

### 3.2. Effect of Dietary Intervention on Diet Quality

The changes in the dietary parameters of the DII^®^ following the 6-month dietary intervention are presented in Table 2. At the baseline, the AID group had significantly higher intakes of omega-3 fatty acids (*p* = 0.015), beta-carotene (*p* = 0.002), flavonols (*p* = 0.029), garlic (*p* < 0.001), and thyme/oregano (*p* = 0.009), while the CD group had higher intakes of carbohydrates (*p* = 0.049), omega-6 fatty acids (*p* = 0.023), trans fatty acids (*p* = 0.007), cholesterol (*p* = 0.004), vitamin A (*p* = 0.008), vitamin B6 (0.023), niacin (*p* = 0.001), riboflavin (*p* = 0.002), thiamine (*p* = 0.002), and zinc (*p* = 0.025). Inthe study trial, the AID group participants significantly reduced the intake of pro-inflammatory components, i.e., the intake of energy (*p* < 0.001), carbohydrates (*p* < 0.001), protein (*p* = 0.009), total fat (*p* < 0.001), saturated fatty acids (*p* < 0.001), trans fat (*p* < 0.001), cholesterol (*p* = 0.030), and vitamin B12 (*p* = 0.012). They also reduced the intake of certain anti-inflammatory components, including monounsaturated fatty acids (*p* = 0.110), polyunsaturated fatty acids (*p* = 0.010), n-6 fatty acids (*p* < 0.001), alcohol (*p* < 0.001), vitamin D (*p* = 0.012), and flavonones (*p* < 0.001). However, they significantly increased the intake of some other anti-inflammatory components, namely, n-3 fatty acids (*p* = 0.049), fiber (*p* = 0.002), vitamin A (*p* = 0.020), vitamin E (*p* = 0.015), vitamin C (*p* = 0.002), beta carotene (*p* < 0.001), folate (*p* < 0.001), flavones (*p* < 0.001), green/black tea (*p* = 0.011), garlic (*p* = 0.006), pepper (*p* = 0.006), turmeric (*p* = 0.002), and thyme/oregano (*p* < 0.001). Due to the anti-inflammatory dietary intervention, the anti-inflammatory potential of the diet in the AID group became almost four times lower than the value at the baseline (283.02% increase, *p* = 0.002).

### 3.3. Effects of the Dietary Intervention on Anthropometric and Body Composition Parameters

The changes in body weight and body composition following the 6-month dietary intervention are presented in Table 3. At the baseline, there were no significant differences regarding the anthropometric and body composition parameters between the two monitored dietary groups. By the study’s end, the total body weight, body fat, and visceral fat had decreased significantly in both groups (−7.06%, *p* < 0.001 vs.−6.21%, *p* < 0.001; −12.15%, *p* < 0.001 vs.−14.10%, *p* < 0.001 and −22.29%, *p* < 0.001 vs.−25.43%, respectively). Furthermore, the reduction in skeletal muscle mass was also prominent and practically the same in both groups (−4.34%, *p* = 0.022 vs.−4.38%, *p* = 0.005). Although at the study baseline, an almost equal distribution of BMI categories was observed in the AID and CD groups, by the study’s end, 21.4% of the AID group and 25.6% of the CD group participants were distributed into the overweight class, while the number of participants with severe obesity reduced by half in the AID group (14.3% to 7.1%) and by two thirds in the CD group (23.1% to 7.7%) (Table S2, Supplementary Materials). 

### 3.4. Effect of the Dietary Interventions on the Cardiometabolic and Inflammatory Statuses

At the baseline, the CD group participants had significantly higher values of HbA1c (*p* = 0.071), total cholesterol (*p* = 0.028), LDL-C (*p* = 0.031), triglycerides (*p* = 0.008), CMI index (*p* = 0.016), IL-6 (*p* < 0.001), and TNF-α (*p* = 0.001) compared to the AID group (Table 4). After the 6 months of the trial, in the CD group, a significant reduction in fasting plasma glucose (−13.08%, *p* < 0.001), insulin resistance that was assessed using the HOMA-IR index (−36.34%, *p* = 0.002), total cholesterol (−7.71%, *p* = 0.002), and LDL-C (−11.98%, *p* = 0.001) was observed, while the status of glycated hemoglobin remained almost the same (Table 4). The AID group participants increased their HDL-C concentrations more than the CD group participants (10.22%, *p* = 0.058 vs.−0.75%, *p* = 0.073). The CMI index decreased in both study groups but not significantly (−22.73%, *p* = 0.153 vs.−16.90%, *p* = 0.622). 

At the baseline, almost half of the participants in the AID group met the IDF criteria for metabolic syndrome and one-third in the CD group, and following the 6-month dietary intervention, the number of participants fulfilling the criteria for metabolic syndrome decreased by one-third in both groups (Table S2 Supplementary Materials).

Compared with the baseline, the AID group participants significantly increased their serum vitamin D concentration (38.35%, *p* < 0.001), while the CD group participants reduced its level, but not significantly (−10.61%, *p* = 0.135) (Table 4). The biomarkers of inflammation were significantly reduced in both groups (Table 4). The CD group participants reduced their hs-CRP (29.46%, *p* = 0.003 vs. −42.18%, *p* = 0.010) and IL-6 (−18.18%, *p* = 0.013 vs. −26.86%, *p* = 0.002) concentrations slightly more than the AID group participants, while the AID group participants achieved a greater reduction in TNF-α (−34.21%, *p* = 0.002 vs.−10.47%, *p* < 0.001). 

### 3.5. Associations Between the Inflammatory Potential of the Diet and the Anthropometric, Cardiometabolic, and Inflammatory Parameters

The linear regression analysis of the participants’ anthropometric, cardiometabolic, and inflammatory parameters after 6 months of the dietary intervention as dependent variables and changes in DII^®^ scores as an independent variable (Table 5) showed a statistically significant negative association only with IL-6 (β = −0.46, 95% CI = −0.56 to 0.46, *p* = 0.026) among the AID group participants. Among the CD group participants, a statistically significant negative association with insulin (β = −0.55, 95% CI = −0.92 to −0.18, *p* = 0.005), HOMA-IR (β = −0.49, 95% CI = −0.88 to −0.11, *p* = 0015), triglycerides (β = −0.56, 95% CI = −0.93 to −0.20, *p* = 0.004), and the CMI index (β = −0.59, 95% CI = −0.94 to −0.23, *p* = 0.003) were observed. There were no other statistically significant associations.

## 4. Discussion

In the present study, the effects of two energy-restricted dietary patterns on the anthropometric, cardiometabolic, and inflammatory parameters of participants with obesity were assessed. Both intervention dietary patterns, described elsewhere [22], had characteristics of the Mediterranean diet, which has been shown to have numerous and significant health benefits, including the prevention of obesity-related diseases [20,38,39,40,41], as well as weight regulation [16,42,43,44]. As the Mediterranean diet has been acknowledged for having anti-inflammatory properties [45], a more frequent and higher intake of foods with anti-inflammatory characteristics was promoted more among the AID group participants. However, a recently published Cochrane systematic review reported that there is still some uncertainty regarding the effects of a Mediterranean-style diet on clinical outcomes and cardiovascular risk factors [46], concluding that the ongoing studies may provide more solid evidence. The study data could advance current scientific knowledge in the field of inflammation and diet, provide guidelines for obesity management, and find its application in routine clinical practice.

### 4.1. The Effect of the Dietary Interventions on Diet Quality Changes

At the study baseline, the average energy intake of the participants in both groups was higher than recommended for their gender, age, and level of physical activity as a result of higher consumption of animal-origin foods and their products, industrially processed cereals, sweets, sweetened beverages, and added fats than recommended, which is in line with Croatian studies on diet quality [27,47,48,49,50,51]. Increased consumption of the above-mentioned food is significantly associated with weight gain, obesity, obesity-related complications, and low-grade chronic inflammation [10,12]. The applied diets in both groups can be considered to be high-protein diets [52] because they reach or exceed 20% of the total energy intake. In the short term, a simultaneously high-protein and low-carbohydrate diet is recommended as a method for weight loss [52]. However, because dietary patterns with this macronutrient ratio generally contain a high proportion of animal-origin foods and saturated fats, which have a significant effect on metabolism and intestinal health, they are not recommended for long-term use [52]. Therefore, in this study, the higher protein content was achieved by a higher intake of fish, legumes, low-fat dairy products, and nuts, and by reducing the intake of meat and their products, as well as sweets, the negative impact of saturated fats was mitigated. By increasing the intake of vegetables and adapting the intake of fruits, participants achieved a change in carbohydrate and fiber intake that is aligned with the characteristics of the Mediterranean and anti-inflammatory diets. Using the DII^®^ to assess the inflammatory potential of the diet, the average diet of both groups at baseline had a minor anti-inflammatory potential due to the consumption of fruits, vegetables, and fish within the recommended intake. These foods contain nutrients that are associated with a significant reduction in low-grade inflammation [10,12]. During the trial, the AID group participants significantly reduced the intake of grains and their products, milk and dairy products, meat and their products, potatoes, sweets and desserts, and alcoholic and non-alcoholic sweetened beverages, which enabled the significant reduction in energy intake, saturated fats, cholesterol, and carbohydrates, which are all acknowledged as being pro-inflammatory [26]. The CD group also made significant reductions in the above-mentioned food items but in minor quantities. 

All participants significantly increased their intake of total flavonoids but significantly reduced the intake of flavan-3-ol and flavonols due to their reduced consumption of certain types of fruits, beans, fruit juices, teas, and desserts rich in cocoa. By significantly raising the intake of green leafy and cruciferous vegetables, green tea, and various herbs and spices, and changing the fruit types, the AID group participants significantly raised the intake of beta-carotene and various phenolic substances, especially the intake of flavone. Higher consumption of food, herbs, and spices expressing antioxidant and anti-inflammatory properties may have preventive and/or therapeutic effects on cardiovascular diseases, neurodegenerative disorders, cancer, and obesity [53,54,55]. The CD group participants also significantly increased the intake of vegetables, spices, and herbs that are typical of the Mediterranean diet, and reduced the intake of fruits, which significantly increased the intake of flavones and other phenolic substances, such as stilbenes and lignans. So far, the intake of various phenolic substances has not been investigated in Croatia, except for flavonoids intake in a cross-sectional study conducted in the same part of Croatia as the present study [27]; thus, this study provides valuable information on the average intake of phenolic substances at both the baseline and the trial’s end. Compared to the mentioned Croatian study [27], the baseline average intake of all phenolic substances by both dietary groups was higher than its average intake in the abovementioned Croatian study. 

Furthermore, this study is unique in Croatia by providing the average intake data of different spices and herbs, because so far, no data on their intake in Croatia have been recorded. The European population consumes on average about 0.5 g of spices per day [56,57]. The intake of herbs and spices is thought to have positive health effects toward preventing or alleviating chronic diseases, such as cardiovascular disease, cancer, arthritis, and neurodegenerative disorders, due to the high content of phenolic substances or their metabolites, which all act on specific receptors or enzymes involved in various anti-inflammatory pathways or immune responses [58]. At the study baseline, the AID group participants consumed almost twice as many spices as the CD group participants, which is three times higher than the average of the European population. At the end of the study, both groups consumed all spices and herbs around three times more than at baseline, especially garlic and Mediterranean spices. The AID group participants doubled their intake of pepper and tripled their intake of turmeric, while at the end of the study, the consumption of turmeric was almost twice as much as the global average [26]. The CD group participants significantly increased their intake of pepper and decreased their intake of turmeric. Since curcumin, the active substance in turmeric, has very low oral bioavailability due to its poor solubility, low absorption, rapid metabolism, and systemic elimination [59], the simultaneous intake of pepper and turmeric can increase its bioavailability by 154% [60]. Turmeric, or curcumin, has been very well researched due to its many favorable biological and health effects, including antioxidant, anti-inflammatory, and anti-tumor activity [59]. Furthermore, curcumin may have a potential benefit in weight management, as it inhibits angiogenesis, adipogenesis, and reduces the fat accumulation in mature adipocytes in a mouse model, with a consequent reduction in adipose tissue and weight gain in mice [59]. The AID group participants significantly increased the anti-inflammatory potential of their diet by increasing the intake of garlic and turmeric, as did the CD group participants by increasing their intake of garlic and pepper. 

At the baseline, both groups had a diet with mild anti-inflammatory potential due to higher consumption of foods containing nutrients with anti-inflammatory effects, namely, fruits, vegetables, and fish. One-third of all participants had an average diet with pro-inflammatory potential, which was significantly reduced by the change in diet quality, which was mainly due to reduced energy and macronutrient intake, and the significant increase in intake of nutrients with anti-inflammatory potential. Most studies that have used the change in DII^®^ as a tool to assess the inflammatory potential of a diet were observational or retrospective, and till now, there is limited evidence from intervention trials to support the claim that a reduction in DII^®^ scores through dietary changes leads to improvements regarding inflammation or related health risk factors [61,62,63,64]. The change in the anti-inflammatory potential of the diet in the AID group toward higher anti-inflammatory potential was somewhat smaller compared to similar studies [63,65]. The greater change in DII^®^ observed in these studies may reflect the higher pro-inflammatory potential of the diet at baseline in comparison to our study. However, our study outcomes showed comparable changes relative to the mentioned studies. The reduction in the DII^®^ scores achieved by the AID group was similar to that reported by an Australian study [62]. The authors of another study evaluating the impact of a 12-month inflammation management intervention on the DII^®^, inflammation, and lipids explained the similar changes in DII^®^ as being due to the reduced number of contacts with study participants, and therefore a lower degree of compliance with dietary recommendations [63]. In our study, a rather mild change in the anti-inflammatory potential of the diet could be explained in terms of a moderate change in the quantity of the nutrients with ahigher anti-inflammatory potential as part of the baseline diet.

### 4.2. The Effect of the Dietary Interventions on the Anthropometric and Body Composition Parameters

As expected, in the present study, the energy-reduced dietary interventions led to significant weight reduction and body composition changes in both groups. Both groups similarly, and significantly, compared to baseline, decreased their weight, BMI, waist circumference, the amount of total and visceral adipose tissue, and skeletal muscle mass. Most of the CD group participants reduced their weight by up to 5%, while most of the AID group participants reduced their weight in the range of 6 to 10%. On average, all participants in this study reduced their weight and BMI by about 7%, which was slightly better than a similar six-month Croatian weight management study, where participants lost on average 10% of their initial body weight and 4% of their BMI [65]. However, the aforementioned study included more participants, and besides a low-fat dietary intervention (<30% of total fats) and pharmacological treatment with orlistat, the participants were encouraged to increase their level of physical activity. Furthermore, an average reduction in body weight, waist circumference, and BMI observed in both treatment arms of the present study was more pronounced than that reported in the five controlled clinical trials that evaluated the Mediterranean diet for obesity management [16]. In comparison to the results of a weight regulation study for the control of polycystic ovary syndrome that used a Mediterranean-inspired low glycemic load anti-inflammatory diet [37] while engaging in physical activity, our participants achieved very similar changes in the body composition. However, the visceral fat mass reduction was greater in our study, while the total fat mass reduction was more noticeable in the aforementioned study [37]. Nevertheless, it is important to underline that, unlike the other weight management studies, our participants were counseled not to engage in additional physical activity to facilitate the interpretation of the effect of the applied diet on the observed changes in body weight and composition.

### 4.3. Effect of the Dietary Interventions on the Cardiometabolic and Inflammatory Statuses

A recent review showed that moderate weight loss (between 5 and 10% of one’s initial body weight) resulted in a significant reduction in glucose, triglycerides, and total cholesterol and improved insulin sensitivity by between 30 and 60%, which are effects greater than those seen with insulin-sensitizing drugs [66]. In the CD group, a significant reduction in fasting plasma glucose, insulin resistance that was assessed using the HOMA-IR index, and total cholesterol and LDL-C was observed, while the level of HbA1c remained almost the same. On the other hand, the AID group participants significantly increased their HDL-C, while the reductions in their fasting plasma glucose, HbA1c, HOMA-IR index, total cholesterol, and LDL-C were not significant. These results may be explained at least in part by the fact that the participants in the AID group had slightly higher levels of fasting glucose, insulin, HOMA-IR index, and HbA1c at baseline than the participants in the CD group. Although HbA1c remains the standard clinical marker of glycemic control, glycemic variability (GV) is an emerging target for blood glucose control and an independent risk factor for diabetes complications [67]. Additionally, GV can be influenced by several nutritional factors, including carbohydrate quality, quantity, and distribution, as well as protein and fiber intake. These factors have important implications for clinical nutrition practice and diabetes management. Due to financial constraints, this kind of measurement could not have been applied in the present study. However, it would be appealing to obtain such data in future research. Nonetheless, the AID group participants achieved a greater decrease in HbA1c level than that reported in the meta-analyses of the Mediterranean diet as a glycemic control diet [68,69,70]. Additionally, concerning comparable studies [20,37,65,71], reductions in the other glycemic parameters were similar.

The AID group participants significantly increased the proportion of total energy intake from fat, reaching 44%. If it exceeds 35–40%, it may affect insulin sensitivity [72]. However, total fats are the sum of different types of fatty acids, and the type of fatty acid is more important than their total amount [72]. The AID group significantly increased the intake of mono- and polyunsaturated fatty acids, particularly omega-3 fatty acids, and did not significantly decrease the level of glycated hemoglobin, which is similar to the results of the PREDIMED (Prevención con Dieta Mediterránea) sub-study [73], where dietary fat intake, including monounsaturated and omega-3 fatty acids, was associated with a higher risk of hyperglycemia. Dyslipidemia has been identified as a risk factor for cardiovascular disease and has been associated with hyperinsulinemia [74]. High concentrations of LDL-C and triglycerides are associated with a risk of cardiovascular disease, while HDL-C levels are the second substitute marker for other cause-and-risk factors of cardiovascular disease [75]. For these reasons, the focus on lowering only one of these biomarkers via dietary changes may affect the intake of certain food components and individuals may be deprived of its pleiotropic effects of the avoided foods, such as dairy food, which contains saturated fatty acids. The AID group participants significantly increased their HDL-C while the CD group participants achieved a significant reduction in their total cholesterol and LDL-C. This could be the effect of a significant reduction in total dietary fat intake and changes in the dietary fatty acid type. While participants in both treatment groups significantly reduced the proportion of total energy intake from saturated fatty acids, the AID group participants significantly increased their proportion of mono- and polyunsaturated fatty acids. Greater reductions in serum triglycerides, total cholesterol, and LDL-C were observed in both treatment arms of the present study than those reported in meta-analyses on the evidence of the efficacy of the Mediterranean diet for the primary prevention and control of cardiovascular diseases [46,71], and somewhat smaller compared to the results obtained by other similar studies [20,37,65,71]. However, the AID group achieved a greater increase in HDL-C concentration than that observed in similar studies [20,37] and the aforementioned meta-analyses [46,71].

Wakabayashi and Daimon created the CMI by placing the ratio of triglycerides to HDL-C in a product with a waist-to-height ratio [36]. This index has been shown to be a good predictor of coronary artery disease and central components of metabolic syndrome [36]. Since most components of the CMI are the components of metabolic syndrome, a change in CMI is associated with changes in metabolic syndrome components. The AID group achieved a greater decrease in CMI than the CD group. This effect was particularly pronounced in the subgroup of participants with metabolic syndrome, who achieved the largest decrease in triglycerides, but also an increase in HDL-C and a greater decrease in total and visceral fat mass compared to the subgroup of participants without metabolic syndrome (data not shown). In the CD group, the largest decrease in CMI was achieved in the subgroup with class I obesity, who achieved the largest decrease in total and visceral fat mass, total cholesterol, and LDL-C. The results presented herein support the fact that the moderate weight loss in individuals with obesity was associated with a reduction in visceral adipose tissue, which in turn led to significant improvements in metabolic syndrome parameters and a reduced risk of coronary heart disease and type 2 diabetes [76] since, in our study, the number of participants fulfilling the criteria for metabolic syndrome decreased by one-third in both groups following the 6-month dietary intervention. Weight loss in individuals with obesity caused by an energy-restricted Mediterranean diet did not significantly increase serum vitamin D levels, as it was obtained with an energy-restricted ketogenic diet, where for every kilogram of body weight lost, serum vitamin D levels tripled after one year of adherence to diet [77]. Participants who followed a ketogenic diet achieved a significantly greater weight loss than those who followed a Mediterranean diet, which has been explained by a lower adherence to the Mediterranean diet [77]. However, the AID group in our study significantly increased their serum vitamin D, which could be explained by an adequate intake of vitamin D from food and supplements. These findings require further investigation regarding the effect of vitamin D supplementation on weight loss.

Diet could be a relatively mild anti-inflammatory intervention, and if investigated in conditions of low-grade inflammation, it may cause mild changes that are difficult to detect [78], especially if weight loss is achieved at the same time. In studies evaluating the association between diet and inflammation, the most disruptive factor is weight loss [79], as inflammation status improvement in response to diet is shown to be dependent on weight loss [79,80]. Because weight loss could affect adipose tissue function and the inflammatory response, a recent review of the effects of whole foods and dietary patterns on circulating inflammatory markers focused on weight-stable adults with overweight and obesity [78]. Although foods and dietary patterns discussed in this review were not found to have significant effects on inflammatory markers in weight-stable individuals, it was difficult to distinguish beneficial or neutral effects because of the methodological inconsistencies between the included studies. Headlend et al. [81] showed that a weight loss of 5% enables a reduction in biomarkers of cardiovascular disease risk, including inflammatory biomarker CRP. A 10% reduction in body weight can decrease serum inflammatory markers by more than twofold and reduce risk factors for cardiovascular disease [81]. In the present study, the biomarkers of inflammation were significantly reduced in both groups, similarly or slightly greater in comparison to some other studies that evaluated the impact of diet on markers of inflammation [78,79]. However, the CD group participants reduced their hs-CRP and IL-6 concentrations slightly more than the AID group participants, while the AID group participants achieved a greater reduction in TNF-α. Both study groups achieved an equal reduction in dietary energy intake, causing a significant loss of total body weight and fat mass. The AID group reduced their total body weight slightly more than the CD group, while the reduction in total and visceral fat mass was slightly more pronounced in the CD group. It has been observed that the reduction of adipose tissue, particularly of visceral adipose tissue, in individuals with obesity, leads to decreased cytokine production [79]. In the present study, the reduction in biomarkers of inflammation could be more of a cause of reducing the total and visceral fat mass than the effect of food ingredients with anti-inflammatory properties. This is in line with review studies of the effects of weight loss and/or diet on the reduction of inflammation [78,79], which concluded that a reduction in total body weight and fat mass following an energy-restricted diet has a significant effect on the reduction in inflammatory markers, while a diet with anti-inflammatory ingredients has only a slight effect on reducing inflammatory markers in weight-stable individuals. Regression analyses of our data showed that changing the diet into a diet with more anti-inflammatory potential was significantly associated only with lower values of IL-6 among the AID group. Other parameters showed a downward association without reaching statistical significance, which could be explained with better-controlled cardiometabolic parameters among the AID group participants than among the CD group. A significant association of lower DII^®^ with lower values of insulin, HOMA-IR, triglycerides, and CMI index was observed among the CD group participants, which was similar to that reported by an Australian study evaluating the effect of a Mediterranean diet on patients with coronary heart disease [62].

### 4.4. Strengths and Limitations

Dietary interventions for obesity management in randomized clinical trials are generally less effective compared to pharmacological interventions or surgery. It is difficult to highlight the effective nutrient(s) of an applied diet due to its complex and still not yet fully investigated metabolism, as well as methodological difficulties regarding standardizing a diet and fully monitoring what participants consume [82]. Choosing an inappropriate period to reveal a clinical effect could be a study limitation [78]. To overcome these limitations, the length of the present study was set to be 6 months, which is consistent with similar dietary intervention trials [20,37,65,71,77]. Significantly more women participated in our study, which also could represent a study limitation regarding data interpretation, as study results could more refer to the female gender rather than males. Participant recruitment was done in an obesity outpatient clinic where more women applied more and were also more willing to be involved in the trial compared to men. Low levels of participant compliance in nutrition trials decrease the power to detect effects on specified endpoints, result in false-negative findings, and ultimately mean that the study is unable to provide evidence to support a potentially beneficial effect of an intervention. Therefore, to maximize and assess compliance with dietary interventions, detailed dietary instructions, frequent contacts with a dietitian, and controlling food diaries according to the specified recommendations using two diet-monitoring methods were provided. This study was also strengthened by detailed assessments of dietary intake that were clarified by the dietitian and comprehensive outcome measures, including the DII^®^, which was not only confirmed as an effective tool for assessing the diet quality, but also for monitoring the change in diet toward a more anti-inflammatory diet.

## 5. Conclusions

An energy-restricted anti-inflammatory diet was shown to be effective in the management of obesity in younger adults. Significant reductions in body weight, BMI, total and visceral adipose tissue, and improvements in body composition, cardiometabolic, and inflammatory parameters were achieved, not only in the intervention anti-inflammatory diet group but also in the control group; this was done by applying the standard protocol for weight reduction based on the Mediterranean diet, which has been acknowledged as an anti-inflammatory diet per se. There are still few data that have been obtained from randomized clinical trials on the effects of dietary interventions on body composition and the metabolic complications of obesity; therefore, the results presented herein could advance current scientific knowledge in the field of inflammation, nutrition, and obesity management.

## Figures and Tables

**Table 1:** Socio-demographic characteristics of the participants (*n* = 81).

Variable	Anti-inflammatory Diet (AID) Group (42 (51.9))	Control Diet (CD) Group (39 (48.1))	*p*-Value
Gender, Men	3 (7.1)	4 (10.3)	0.619 ^a^
Women	39 (92.9)	35 (89.7)	
Age (years) (Mean± SD)	43.60 ± 5.75	41.72 ± 6.70	0.178 ^b^
Marriage Status, Single	3 (7.1)	3 (7.7)	0.537 ^a^
Married	38 (90.5)	33 (84.6)	
Divorced	1 (2.4)	3 (7.7)	
Widowed	0 (0)	0 (0)	
Education Degree, Elementary school	0 (0)	2 (5.1)	0.00 ^a^
High School	12 (28.6)	22 (56.4)	
University	29 (69.0)	15 (38.5)	
Postgraduate	1 (2.4)	0 (0)	
Professional Status, Unemployed	1 (2.4)	9 (23.1)	0.033 ^a^
Employed	40 (95.2)	30 (76.9)	
Student	1 (2.4)	0 (0)	
Physical Activity, Inactive	15 (35.7)	18 (46.2)	0.315 ^a^
Moderately Inactive	9 (21.4)	3 (7.7)	
Moderately Active	14 (33.3)	15 (38.5)	
Active	4 (9.5)	3 (7.7)	
Obesity, First Degree	26 (61.9)	21 (53.8)	0.584 ^a^
Second Degree	10 (23.8)	9 (23.1)	
Third Degree	6 (14.3)	9 (23.1)	
Metabolic Syndrome, Yes	20 (47.6)	13 (33.3)	0.191 ^a^
Autoimmune Thyroid Disease	10 (23.8)	3 (7.7)	0.048 ^a^

Data are presented as number (%) unless otherwise stated. ^a^ *p* < 0.05 was tested using the χ^2^ test for independent samples. ^b^ *p* < 0.05 was tested with Student’s *t*-test for independent samples.

**Table 2:** The changes in the dietary parameters of the DII^®^.

Variable	Anti-inflammatory Diet Group				Control Diet Group					
	Baseline	Trial End	Change (%)	*p*-Value ^c^	Baseline	Trial End	Change (%)	*p*-Value ^c^	Baseline *p*-Value ^d^	Trial End *p*-Value ^e^
Energy (MJ)	10.03 (2.64)	6.92 (0.47)	−31.01	<0.001	11.21 (2.59)	7.63(0.40)	−31.94	<0.001	0.129	<0.001
Protein (g)	103.03 (31.08)	85.60 (14.42)	−16.92	0.009 ^a^	113.70 (26.49)	97.13 (8.72)	−14.57	0.001 ^a^	0.211 ^a^	0.002 ^a^
Carbohydrate (g)	233.91 (77.56)	145.35 (31.43)	−37.86	<0.001 ^a^	280.21 (79.75)	172.96 (17.32)	−38.27	<0.001 ^a^	0.049 ^a^	<0.001 ^a^
Fat (g)	111.94 (27.14)	81.05 (13.91)	−27.60	<0.001 ^a^	120.07 (30.28)	80.69 (9.58)	−32.80	<0.001 ^a^	0.338 ^a^	0.918 ^a^
MUFA (g)	44.93 (11.90)	39.30 (8.69)	−12.53	0.110 ^a^	43.93 (10.30)	32.39 (3.68)	−26.27	<0.001 ^a^	0.759 ^a^	<0.001 ^a^
PUFA (g)	19.02 (5.39)	15.40 (3.63)	−19.03	0.010 ^a^	21.10 (4.92)	13.26 (1.24)	−37.16	<0.001 ^a^	0.174 ^a^	0.009 ^a^
Omega-3 (g)	1.16 (0.68)	1.27 (0.54)	9.48	0.049 ^a^	0.79 (0.21)	0.54 (0.15)	−31.65	<0.001 ^a^	0.015 ^a^	<0.001 ^b^
Omega-6 (g)	0.73 (0.21)	0.47 (0.19)	−35.62	<0.001 ^a^	0.91 (0.30)	0.60 (0.12)	−3.23	0.159 ^a^	0.023 ^a^	<0.001 ^a^
Saturated fat (g)	41.50 (14.69)	20.20 (3.10)	−51.33	<0.001 ^a^	49.35 (14.67)	28.63 (3.56)	−41.99	<0.001 ^a^	0.074 ^a^	<0.001 ^a^
Trans fat (g)	1.67 (0.41)	1.32 (0.62)	−20.96	<0.001 ^a^	2.13 (0.67)	1.31 (0.30)	−38.50	<0.001 ^a^	0.007 ^a^	0.941 ^a^
Cholesterol (mg)	380.81 (160.38)	318.51 (175.61)	−16.36	0.030 ^a^	477.55 (463.01)	463.01 (125.57)	−3.04	0.643	0.004 ^a^	<0.001 ^a^
Fiber (g)	27.44 (11.30)	33.90 (5.22)	23.54	0.002 ^a^	25.46 (6.69)	28.69 (3.95)	12.69	0.146	0.467 ^a^	<0.001 ^a^
Alcohol (g)	6.10 (12.29)	0.21 (1.02)	−96.56	<0.001 ^b^	3.34 (3.87)	2.44 (3.33)	−26.95	0.605 ^b^	0.991 ^b^	0.005 ^b^
Caffeine (g)	315.41 (162.30)	239.48 (112.49)	−24.07	0.675 ^a^	218.35 (221.56)	202.07 (79.48)	−7.46	0.864 ^b^	0.095 ^a^	0.193 ^a^
Folate (μg)	433.81 (243.26)	539.07 (158.45)	47.86	<0.001 ^a^	330.28 (82.88)	373.58 (53.67)	11.27	0.042 ^a^	0.055 ^a^	<0.001 ^a^
Beta carotene (μg)	4.20 (2.16)	5.72 (1.95)	36.19	<0.001 ^b^	3.75 (1.57)	3.32 (0.71)	−11.47	<0.001 ^a^	0.002 ^b^	0.193 ^a^
Vitamin A (RE)	700.87 (334.55)	919.41 (570.63)	31.18	0.020 ^a^	969.03 (530.92)	1083.97 (540.80)	11.86	0.190 ^b^	0.008 ^a^	0.187 ^a^
Vitamin B6 (mg)	2.24 (0.90)	2.42 (1.16)	0.00	0.480 ^a^	2.81 (1.31)	2.00 (0.70)	−28.83	0.022 ^b^	0.024 ^a^	0.250 ^b^
Vitamin B12 (μg)	3.14 (1.33)	2.46 (1.44)	−21.66	0.012 ^a^	3.18 (1.07)	2.30 (0.47)	−27.67	<0.001 ^a^	0.884 ^a^	0.139 ^a^
Vitamin C (mg)	204.13 (153.16)	287.64 (106.32)	40.91	0.002 ^a^	166.32 (76.80)	133.05 (33.06)	−20.00	0.007 ^a^	0.287 ^a^	<0.001 ^a^
Vitamin D (μg)	14.54 (22.06)	6.24 (17.41)	−57.08	0.012 ^b^	10.10 (11.68)	2.18 (0.96)	−78.42	<0.001 ^a^	0.966 ^b^	0.261 ^a^
Vitamin E (mg)	22.51 (12.98)	26.81 (8.19)	19.10	0.015 ^a^	16.12 (2.97)	12.35 (2.44)	−23.39	<0.0001 ^a^	0.113 ^b^	<0.001 ^a^
Iron (mg)	8.22 (2.05)	8.55 (2.12)	4.01	0.487 ^a^	9.03 (2.81)	9.37 (1.85)	3.77	0.571 ^a^	0.143 ^a^	0.017 ^a^
Magnesium (mg)	412.51 (132.81)	440.18 (80.88)	6.71	0.213 ^a^	393.11 (88.66)	371.74 (36.78)	−5.44	0.080 ^a^	0.557 ^a^	<0.001 ^a^
Niacin (mg)	19.40 (6.37)	17.38 (3.91)	−10.41	0.077 ^a^	24.82 (8.20)	17.29 (1.68)	−30.34	<0.001 ^a^	0.001 ^a^	0.881 ^a^
Riboflavin (mg)	1.60 (0.65)	2.01 (0.71)	25.63	0.089 ^a^	2.07 (0.70)	2.24 (0.31)	8.21	0.189 ^a^	0.002 ^a^	0.056 ^a^
Selenium (μg)	37.42 (17.60)	31.76 (10.95)	−15.13	0.467 ^a^	35.86 (7.79)	39.61 (10.99)	10.46	0.309 ^a^	0.696 ^a^	0.018 ^a^
Thiamine (mg)	1.54 (0.51)	1.73 (0.39)	12.34	0.057 ^a^	1.93 (0.58)	1.56 (0.25)	−19.17	0.001 ^a^	0.002 ^a^	0.023 ^a^
Zinc (mg)	15.42 (4.59)	13.91 (3.17)	−9.79	0.084 ^a^	18.12 (4.85)	13.70 (1.30)	−24.39	<0.001 ^a^	0.025 ^a^	0.772 ^a^
Flavan-3-ol (mg)	28.81 (26.79)	15.83 (10.41)	−45.05	0.056 ^b^	23.37 12.83	8.34 (2.39)	−64.31	<0.001 ^a^	0.807 ^b^	<0.001 ^b^
Flavones (mg)	2.90 (2.03)	5.58 (3.40)	92.41	<0.001 ^a^	2.27 (1.40)	3.14 (1.46)	38.33	0.037 ^b^	0.366 ^b^	0.002 ^a^
Flavonols (mg)	147.41 (78.61)	149.40 (6.79)	1.35	0.856 ^a^	107.57 (34.82)	74.19 (39.99)	−31.03	<0.001 ^a^	0.029 ^a^	<0.001 ^a^
Flavonones (mg)	46.08 (37.87)	22.96 (22.86)	−50.17	<0.001 ^a^	65.59 (74.68)	3.64 (3.98)	-94.45	<0.001 ^a^	0.856 ^b^	<0.001 ^b^
Anthocyanidins (mg)	24.25 (34.64)	29.97 (28.37)	23.59	0.406 ^a^	15.54 (9.33)	24.30 (19.43)	56.37	0.606 ^a^	0.873 ^b^	0.426 ^a^
Isoflavones (mg)	0.36 (0.22)	0.56 (0.28)	55.55	0.864 ^a^	0.27 (0.36)	0.62 (0.49)	129.63	0.787 ^a^	0.816 ^a^	0.374 ^a^
Eugenol (mg)	0.03 (0.06)	0.04 (0.12)	33.33	0.499 ^b^	0.02 (0.02)	0.00 (0.01)	−100.00	0.004 ^b^	0.062 ^b^	0.262 ^b^
Green/black tea (g)	1.07 (0.91)	2.86 (1.66)	167.29	0.011 ^a^	0.96 (0.24)	2.08 (1.84)	116.67	0.009 ^a^	0.358 ^a^	0.686 ^a^
Garlic (g)	2.10 (1.49)	6.60 (2.28)	86.57	0.006 ^b^	0.75 (1.19)	4.32 (1.44)	476.00	<0.001 ^b^	<0.001 ^a^	<0.001 ^a^
Ginger (g)	0.15 (0.38)	0.19 (0.26)	26.67	0.098 ^b^	0.06 (0.07)	0.00 (0.00)	−100.00	<0.001 ^b^	0.481 ^b^	<0.001 ^b^
Onion (g)	14.61 (7.03)	13.11 (10.63)	−10.27	0.074 ^a^	17.97 (9.01)	10.89 (7.89)	−39.40	0.009 ^b^	0.163 ^a^	0.418 ^a^
Pepper (g)	0.67 (0.68)	1.25 (0.56)	86.57	0.006 ^b^	0.55 (0.54)	0.70 (0.23)	27.27	0.033 ^a^	0.621 ^b^	<0.001 ^a^
Rosemary (mg)	0.11 (0.17)	0.17 (0.29)	54.55	0.909 ^a^	0.02 (0.03)	0.01 (0.01)	−50.00	<0.001 ^b^	0.139 ^b^	<0.001 ^b^
Saffron (g)	0.01 (0.01)	0.01 (0.02)	0.00	0.939 ^b^	0.00 (0.00)	0.01 (0.01)	0.00	0.999 ^b^	0.917 ^b^	0.884 ^b^
Turmeric (mg)	0.38 (0.94)	1.05 (0.69)	176.32	0.002 ^b^	0.08 (0.18)	0.01 (0.03)	−87.50	0.002 ^b^	0.220 ^b^	<0.001 ^b^
Thyme/oregano (mg)	0.09 (0.11)	0.29 (0.16)	222.22	<0.001 ^a^	0.04 (0.08)	0.13 (0.06)	225.00	0.002 ^b^	0.009 ^b^	0.001 ^b^
DII^®^	−0.53 (2.29)	−2.03 (0.97)	283.02	0.002 ^a^	−0.23 (1.28)	−0.31 (1.01)	30.43	0.725 ^a^	0.579 ^a^	<0.001 ^a^

Data are presented as the mean (SD). ^a^ *p* < 0.05 was tested using Student’s *t*-test for dependent samples. ^b^ *p* < 0.05 was tested using the Wilcoxon z-test for dependent samples. ^c^ Comparison within dietary groups (baseline and after 6 months). ^d^ Baseline differences between the AID and CD groups. ^e^ Differences after 6 months between the AID and CD groups. PUFA, polyunsaturated fatty acids; MUFA, monounsaturated fatty acids; RE, retinol equivalent;DII^®^, Dietary Inflammatory Index. The CD group participants also significantly reduced the intake of pro-inflammatory components, the intake of energy (*p* <0.001), carbohydrates (*p* < 0.001), protein (*p* = 0.002), total fat (*p* < 0.001), saturated fatty acids (*p* < 0.001), trans fat (*p* < 0.001), and vitamin B12 (*p* < 0.001). The intake of certain anti-inflammatory components, such as monounsaturated fatty acids (*p* < 0.001), polyunsaturated fatty acids (*p* < 0.001), n-3 fatty acids (*p* < 0.001), vitamins B6 (*p* = 0.022), vitamin C (*p* = 0.007), vitamin D (*p* < 0.001), vitamin E (*p* < 0.001), niacin (*p* < 0.001), thiamine (*p* < 0.001), zinc (*p* < 0.001), flavan-3-ol (*p* < 0.001), flavonols (*p* < 0.001), flavonones (*p* < 0.001), eugenol (*p* = 0.004), ginger (*p* < 0.001), rosemary (*p* < 0.001), and turmeric (*p* < 0.001), were reduced, while the intake of other anti-inflammatory components, such as folate (*p* < 0.001), flavones (*p* = 0.037), green/black tea (*p* = 0.009), garlic (*p* < 0.001), pepper (*p* = 0.033), and thyme/oregano (*p* < 0.001), were significantly increased. The anti-inflammatory potential of the diet in the CD group increased bythe end of the trial, although not significantly (30.43%, *p* = 0.725). By significantly reducing the intake of grains and products (−42.56%, *p* < 0.001), potatoes (−62.34%, *p* < 0.001), sweets (−90.85%, *p* < 0.001), and juices and sweetened beverages (−100.00%, *p* < 0.001), the AID group participants significantly reduced the proportion of total energy intake from carbohydrates (−8.58%, *p* < 0.001) (Tables S1 and S3, Supplementary Materials. The shift in consumption toward the higher intake of nuts (54.25%, *p* < 0.001), legumes (47.50%, *p* = 0.094), and eggs (27.25%, *p* = 0.314) significantly raised the proportion of the total energy intake from proteins (20.16%, *p* < 0.001) (Tables S1 and S3, Supplementary Materials). Consequently, the proportions of energy intake from mono- (26.75%, *p* < 0.001) and polyunsaturated fatty acids (17.37%, *p* = 0.030), particularly n-3 fatty acids (56.82%, *p* < 0.001), were increased, while those from saturated fats (−29.46%, *p* < 0.001) and omega-6 fatty acids (−3.70%, *p* = 0.003) were significantly reduced (Table S3, Supplementary Materials). Similar changes were observed among CD group participants, but not in the intake of monounsaturated fats (Tables S1 and S3, Supplementary Materials).

**Table 3:** The changes in the anthropometric and body composition parameters.

Variable	Anti-inflammatory Diet Group				Control Diet Group					
	Baseline	Trial End	Change (%)	*p*-Value ^c^	Baseline	Trial End	Change (%)	*p*-Value ^c^	Baseline *p*-Value ^d^	Trial End *p*-Value ^e^
Body weight (kg)	102.94 (14.19)	95.67 (11.72)	−7.06	<0.001 ^a^	101.35 (21.93)	95.06 (21.36)	−6.21	<0.001 ^a^	0.770 ^a^	0.903 ^a^
Body mass index (kg/m^2^)	35.36 (4.27)	32.90 (3.90)	−6.96	<0.001 ^a^	33.40 (5.48)	30.99 (4.32)	−7.22	<0.001 ^a^	0.179 ^a^	0.119 ^a^
Waist circumference (cm)	108.43 (8.37)	102.91 (7.77)	−5.09	<0.001 ^a^	107.86 (10.11)	100.88 (10.04)	−6.47	<0.001 ^a^	0.482 ^b^	0.442 ^a^
Fat tissue (kg)	46.49 (10.17)	40.84 (7.87)	−12.15	<0.001 ^a^	45.89 (7.71)	39.42 (8.82)	−14.10	<0.001 ^a^	0.807 ^b^	0.328 ^b^
Fat tissue (%)	44.87 (4.38)	42.34 (4.80)	−5.64	<0.001 ^a^	45.57 (2.57)	42.20 (2.98)	−7.40	0.001 ^a^	0.505 ^a^	0.755 ^a^
Visceral adipose tissue (l)	3.14 (1.32)	2.44 (1.05)	−22.29	<0.001 ^a^	3.46 (1.63)	2.58 (1.37)	−25.43	<0.001 ^a^	0.376 ^b^	0.798 ^b^
Non-fat tissue (kg)	56.45 (6.50)	55.28 (6.09)	−2.07	0.139 ^a^	54.88 (11.50)	53.81 (12.97)	−1.95	0.019 ^b^	0.569 ^b^	0.625 ^a^
Non-fat tissue (%)	55.13 (4.38)	57.80 (4.66)	4.84	<0.001 ^a^	54.40 (2.61)	57.21 (2.40)	5.17	<0.001 ^a^	0.484 ^a^	0.587 ^a^
Skeletal muscle tissue (kg)	27.41 (3.86)	26.22 (3.29)	−4.34	0.022 ^a^	26.96 (6.21)	25.78 (7.34)	−4.38	0.005 ^b^	0.449 ^b^	0.085 ^b^
Total water (L)	42.40 (4.95)	41.33 (4.40)	−2.52	0.009 ^a^	41.16 (8.26)	40.04 (9.50)	−2.72	0.091 ^a^	0.537 ^a^	0.053 ^b^
Total water (%)	41.13 (8.37)	43.17 (4.08)	4.96	<0.001 ^a^	40.34 (1.70)	42.66 (1.81)	5.73	0.009 ^a^	0.407 ^a^	0.582 ^a^

Data are presented as the mean (SD). ^a^ *p* < 0.05 was tested with Student’s *t*-test for dependent samples. ^b^ *p* < 0.05 was tested using the Wilcoxon z-test for dependent samples. ^c^ Comparison within dietary groups (baseline and after 6 months). ^d^ Baseline differences between the AID and CD groups. ^e^ Differences after 6 months between the AID and CD groups.

**Table 4:** The changes in the cardiometabolic and inflammatory parameters.

Variable	Anti-inflammatory Diet Group				Control Diet Group					
	Baseline	Trial End	Change (%)	*p*-Value ^c^	Baseline	Trial End	Change (%)	*p*-Value ^c^	Baseline *p*-Value ^d^	Trial End *p*-Value ^e^
Glucose (mmol/L)	5.69 (1.41)	5.48 (0.62)	−3.69	0.284 ^b^	5.58 (0.45)	4.85 (0.59)	−13.08	<0.001 ^a^	0.107 ^b^	<0.001 ^a^
HbA1c (mmol/mol)	35.26 (6.45)	34.66 (7.62)	−1.70	0.855 ^a^	38.34 (4.87)	38.37 (4.74)	0.08	0.121 ^a^	0.071 ^a^	0.050 ^a^
HbA1c (%)	5.40 (0.63)	5.52 (0.55)	2.22	0.049 ^a^	5.64 (0.45)	5.65 (0.45)	0.18	0.127 ^a^	0.128 ^a^	0.371 ^a^
Insulin (mU/L)	18.22 (11.69)	16.19 (9.98)	−11.14	0.946 ^a^	16.10 (4.91)	11.73 (3.88)	−27.14	0.008 ^b^	0.419 ^b^	0.048 ^a^
HOMA - IR (pmol/L)	4.84 (3.89)	4.09 (3.00)	−15.50	0.307 ^b^	3.99 (1.26)	2.54 (0.86)	−36.34	0.002 ^a^	0.572 ^b^	0.040 ^b^
Total cholesterol (mmol/L)	5.25 (1.10)	4.97 (1.34)	−5.33	0.594 ^a^	5.84 (0.65)	5.39 (0.80)	−7.71	0.002 ^a^	0.028 ^a^	0.193 ^a^
HDL-C (mmol/L)	1.37 (0.21)	1.51 (0.53)	10.22	0.058 ^a^	1.34 (0.20)	1.33 (0.13)	−0.75	0.073 ^a^	0.642 ^a^	0.127 ^b^
LDL-C (mmol/L)	3.30 (1.02)	3.15 (0.99)	−4.55	0.357 ^a^	3.84 (0.61)	3.38 (0.63)	−11.98	<0.001 ^a^	0.031 ^a^	0.343 ^a^
Triglycerides (mmol/L)	1.31 (0.94)	1.15 (0.56)	−12.21	0.446 ^b^	1.50 (0.40)	1.33 (0.47)	−11.33	0.393 ^a^	0.008 ^b^	0.144 ^b^
CMI index	0.66 (0.60)	0.51 (0.39)	−22.73	0.153 ^b^	0.71 (0.21)	0.59 (0.22)	−16.90	0.622 ^a^	0.016 ^b^	0.145 ^b^
Vitamin D (nmol/L)	48.13 (21.36)	66.59 (24.72)	38.35	<0.001 ^a^	54.09 (11.47)	48.35 (18.46)	−10.61	0.135 ^b^	0.237 ^a^	0.006 ^a^
hs-CRP (mg/L)	6.28 (5.48)	4.43 (4.29)	−29.46	0.003 ^a^	6.78 (4.09)	3.92 (0.92)	−42.18	0.010 ^b^	0.311 ^b^	0.662 ^b^
IL-6 (pg/mL)	0.77 (0.63)	0.63 (0.36)	−18.18	0.013 ^a^	1.34 (0.85)	0.98 (0.81)	−26.86	0.002 ^a^	<0.001 ^a^	0.001 ^a^
TNF-α (pg/mL)	0.38 (0.19)	0.25 (0.09)	−34.21	0.002 ^a^	1.72 (0.33)	1.54 (0.36)	−10.47	<0.001 ^a^	0.001 ^b^	<0.001 ^b^

Data are presented as the mean (SD). ^a^ *p* < 0.05 tested with Student’s *t*-test for dependent samples. ^b^ *p* < 0.05 tested with the Wilcoxon z-test for dependent samples. ^c^ Comparison within dietary groups (baseline and after 6 months). ^d^ Baseline differences between the AID and CD groups. ^e^ Differences after 6 months between the AID and CD groups. HbA1c, glycated hemoglobin; HOMA-IR, Homeostatic Model Assessment for Insulin Resistance; HDL-C, high-density lipoprotein cholesterol; LDL-C, low-density lipoprotein cholesterol; CMI, cardiometabolic index; hs-CRP, high-sensitivity C-reactive protein; IL-6, interleukin-6; TNF-α, tumor necrosis factor alpha.

**Table 5:** Regression analyses of the participants’ anthropometric, cardiometabolic, and inflammatory parameters after 6 months of dietary intervention as dependent variables and changes in the DII^®^ scores as independent variables.

Variable	Anti-inflammatory Diet Group				Control Diet Group			
	β	95% CI		*p*-Value	β	95% CI		*p*-Value
Body weight (kg)	−0.13	−0.31	0.58	0.540	−0.21	−0.64	0.22	0.328
Body mass index (kg/m^2^)	−0.19	−0.25	0.69	0.247	−0.33	−0.75	0.09	0.114
Waist circumference (cm)	−0.06	−0.16	0.79	0.087	−0.12	−0.56	0.32	0.582
Fat mass (kg)	−0.18	−0.26	0.70	0.234	−0.26	−0.69	0.16	0.214
Fat free mass (kg)	−0.03	−0.48	0.42	0.897	−0.03	−0.47	0.41	0.899
Skeletal muscle tissue (kg)	0.05	−0.41	0.50	0.831	−0.03	−0.48	0.41	0.875
Visceral adipose tissue (L)	−0.21	−0.23	0.65	0.339	−0.11	−0.33	0.55	0.608
Total water (L)	−0.00	−0.46	0.45	0.990	−0.04	−0.48	0.40	0.853
Glucose (mmol/L)	−0.14	−0.59	0.31	0.525	−0.02	−0.42	0.46	0.932
HbA1c (mmol/mol)	−0.17	−0.28	0.61	0.446	−0.04	−0.34	0.49	0.835
HbA1c (%)	−0.02	−0.44	0.47	0.943	−0.03	−0.44	0.47	0.878
Insulin (mU/L)	−0.04	−0.49	0.42	0.865	−0.55	−0.92	−0.18	0.005
HOMA-IR (pmol/L)	−0.06	−0.52	0.39	0.778	−0.49	−0.88	−0.11	0.015
Total cholesterol (mmol/L)	−0.01	−0.44	0.46	0.961	−0.38	−0.79	0.03	0.071
HDL-C (mmol/L)	−0.05	−0.51	0.40	0.814	0.17	−0.27	0.61	0.428
LDL-C (mmol/L)	−0.10	−0.55	0.35	0.659	−0.22	−0.65	0.21	0.300
Triglycerides (mmol/L)	−0.13	−0.58	0.32	0.565	−0.56	−0.93	−0.20	0.004
CMI index	−0.05	−0.51	0.40	0.804	−0.59	−0.94	−0.23	0.003
Vitamin D (nmol/L)	−0.07	−0.39	0.52	0.766	−0.23	−0.66	0.20	0.277
hs-CRP (mg/L)	−0.06	−0.40	0.51	0.798	−0.21	−0.64	0.22	0.319
IL-6 (pg/mL)	−0.46	−0.56	0.46	0.026	−0.14	−0.58	0.30	0.520
TNF-α (pg/mL)	−0.17	−0.62	0.28	0.440	−0.05	−0.50	0.39	0.804

Models were adjusted for diet (the group of intervention), age, gender, and physical activity. Models were not adjusted for total energy intake because it is one of the DII^®^ components. HbA1c, glycated hemoglobin; HOMA-IR, Homeostatic Model Assessment for Insulin Resistance; HDL-C, high-density lipoprotein cholesterol; LDL-C, low-density lipoprotein cholesterol; CMI, cardiometabolic index; hs-CRP, high-sensitivity C-reactive protein; IL-6, interleukin-6; TNF-α, tumor necrosis factor-alpha.

## Supplementary Materials

The following are available online at https://www.mdpi.com/2072-6643/12/11/3583/s1, Table S1: The changes in the consumption of food items, Table S2: The distribution of participants according to BMI classification and metabolic syndrome at the end of the trial (*n* = 81) (*n* (%)), Table S3: The proportion of total energy intake from dietary components.
