# Supplementary material for: The Efficacy of an Energy-Restricted Anti-Inflammatory Diet for the Management of Obesity in Younger Adults
Source: Nutrients. 2020 Nov 22;12(11):3583. doi: 10.3390/nu12113583 (PMC7700374; doi:10.3390/nu12113583)
Supplement: Supplementary file 1 [file nutrients-12-03583-s001.pdf]

nutrients

MDPI

Supplementary Materials

Table S1. The changes in consumption of food items.

Variable	Anti-inflammatory diet group				Control diet group					
	Baseline	Trial end	Change (%)	P-value[c]	Baseline	Trial end	Change (%)	P-value[c]	Baseline P-value[d]	Trial end P-Value[e]
Cereals and products (g/day)	194.34 (81.05)	111.62 (51.69)	-42.56	<0.001[a]	252.86 (87.46)	231.85 (43.46)	-8.31	0.231[a]	0.029[a]	<0.001[a]
Milk and dairy products (g/day)	380.98 (254.66)	205.01 (100.48)	-46.19	0.002[a]	398.72 (165.03)	388.93 (34.57)	-2.46	0.135[a]	0.777[a]	<0.001[a]
Eggs (g/day)	24.81 (15.96)	31.57 (22.89)	27.25	0.314[a]	37.00 (26.89)	30.26 (27.74)	-18.22	0.199[b]	0.073[b]	0.816[a]
Meat and meat products (g/day)	214.70 (53.78)	180.55 (90.50)	-15.91	0.002[a]	244.65 (74.84)	188.72 (29.93)	-22.86	<0.001[a]	0.124[a]	0.677[a]
Fish (g/day)	73.25 (50.24)	55.92 (42.32)	-23.66	0.732[a]	47.85 (23.82)	69.06 (35.25)	44.33	0.211[a]	0.139[b]	0.253[a]
Legumes (g/day)	36.27 (33.92)	53.52 (47.58)	47.50	0.094[b]	32.87 (15.52)	31.94 (27.28)	-2.83	0.554[a]	0.159[b]	0.062[a]
Vegetables (g/day)	345.14 (249.86)	636.65 (156.24)	84.46	<0.001[b]	229.77 (106.06)	405.56 (102.93)	76.51	<0.001[a]	0.044[b]	<0.001[a]
Potatoes (g/day)	106.33 (67.95)	40.04 (48.05)	-62.34	<0.001[a]	210.18 (119.14)	65.87 (38.49)	-68.66	<0.001[a]	<0.001[a]	0.047[a]
Fruit (g/day)	252.68 (102.91)	241.43 (89.76)	-4.45	0.769[a]	180.13 (105.68)	125.65 (44.87)	-30.24	0.010[a]	0.022[a]	<0.001[a]
Nuts (g/day)	18.10 (24.08)	27.92 (12.87)	54.25	<0.001[a]	9.58 (11.24)	8.58 (6.03)	-10.44	0.078[a]	0.048[a]	<0.001[a]
Desserts (g/day)	65.37 (44.64)	5.98 (8.35)	-90.85	<0.001[a]	104.98 (52.09)	4.14 (14.03)	-96.06	<0.001[b]	0.007[a]	0.002[b]
Added fat (g/day)	11.73 (7.21)	13.64 (5.21)	16.28	<0.001[b]	15.17 (5.08)	8.76 (55.53)	-42.25	0.809[b]	0.064[a]	0.012[a]
Coffee (g/day)	147.83 (76.31)	112.86 (7.92)	-23.66	0.779[a]	101.73 (103.69)	95.31 (4.04)	-6.31	<0.001[a]	0.061[b]	0.195[a]
Fruit juice (ml/day)	35.26 (63.08)	0.00 (23.05)	-100.00	<0.001[a]	88.26 (145.83)	0.00 (37.49)	-100.00	<0.001[b]	0.359[b]	-
Sweetened refreshing drinks (ml/day)	37.33 (84.78)	0.00 (0.00)	-100.00	<0.001[a]	45.94 (48.84)	0.00 (0.00)	-100.00	<0.001[a]	0.583[a]	-
Alcoholic beverages (ml/day)	35.42 (65.06)	1.09 (5.21)	-96.92	<0.001[a]	32.42 (40.64)	40.61 (55.53)	25.26	<0.001[a]	0.539[b]	0.004[b]

Data are presented as the mean (SD). [a] P <0.05 tested with Student t-test for dependent samples. [b] P <0.05 tested with Wilcoxon z-test for dependent samples. [c] Comparison within dietary groups (baseline and after 6 months). [d] Baseline differences between the AID and CD groups. [e] Differences after 6 months between the AID and CD groups.

Table S2. The distribution of participants according to BMI classification and metabolic syndrome at the end of the trial (n=81).

Variable	Anti-inflammatory diet group (42 (51.9))	Control diet group (39 (48.1))	P-value
Overweight	9 (21.4)	10 (25.6)	0.628[a]
Obesity, 1st degree	24 (57.1)	17 (43.6)	
2nd degree	6 (14.3)	9 (23.1)	
3rd degree	3 (7.1)	3 (7.7)	
Metabolic syndrome, Yes	14 (33.33)	13 (13.33)	0.813[a]

Data are presented as number (%).[a] $p < 0.05$ tested with $\chi 2$ test for independent samples.

Table S3. The proportion of total energy intake from dietary components (%MJ)

Variable	*Anti-inflammatory diet group*				*Control diet group*					
	Baseline	Trial end	Change (%)	P-value[c]	Baseline	Trial end	Change (%)	P-value[c]	Baseline P-value[d]	Trial end P-Value[e]
Protein	17.16 (1.70)	20.62 (2.63)	20.16	<0.001[a]	17.07 (1.97)	21.33 (1.86)	24.96	<0.001[a]	0.872[a]	0.006[a]
Carbohydrate	38.59 (6.06)	35.28 (7.71)	-8.58	<0.001[a]	41.60 (4.71)	37.98 (3.72)	-8.70	0.535[a]	0.063[a]	0.131[a]
Fat	42.63 (6.54)	44.01 (6.19)	3.24	0.021[a]	40.28 (3.65)	39.77 (3.69)	-1.27	0.005[a]	0.133[a]	0.292[a]
MUFA	16.86 (4.48)	21.37 (8.69)	26.75	<0.001[a]	14.75 (1.78)	15.98 (3.49)	8.34	0.856[a]	0.018[a]	<0.001[a]
PUFA	7.14 (2.08)	8.38 (2.88)	17.37	0.029[a]	7.09 (0.77)	6.54 (1.18)	-7.76	0.001[a]	0.677[a]	<0.001[a]
Omega-3	0.44 (0.31)	0.69 (0.43)	56.82	<0.001[a]	0.27 (0.09)	0.27 (0.14)	0.00	0.109[a]	0.010[a]	<0.001[a]
Omega-6	0.27 (0.07)	0.26 (0.15)	-3.70	0.003[a]	0.31 (0.07)	0.30 (0.11)	-3.23	0.158[a]	0.210[a]	0.071[a]
Saturated fat	15.58 (2.91)	10.99 (24.62)	-29.46	<0.001[a]	16.58 (2.48)	14.13 (33.84)	-14.78	<0.001[a]	0.199[a]	<0.001[a]
Trans fat	0.63 (0.30)	0.72 (0.49)	14.29	0.279[a]	0.72 (0.15)	0.65 (0.29)	-9.72	0.214[a]	0.363[a]	0.363[a]
Alcohol	1.63 (2.79)	1.63 (2.79)	0.00	0.999[a]	1.05 (1.30)	0.92 (1.26)	-12.38	0.006[a]	0.464[a]	0.037[a]

Data are presented as the mean (SD). a P <0.05 tested with Student t-test for dependent samples. b P <0.05 tested with Wilcoxon z-test for dependent samples. c Comparison within dietary groups (baseline and after 6 months). d Baseline differences between the AID and CD groups. e Differences after 6 months between the AID and CD groups.SFA, Saturated fatty acids; PUFA, Polyunsaturated fatty acids; MUFA, Monounsaturated fatty acids; Omega-3 FA, Omega-3 fatty acids; Omega-6 FA, Omega-6 fatty acids; TFA, Trans fatty acids

© 2020 by the authors. Submitted for possible open access publication under the terms and conditions of the Creative Commons Attribution (CC BY) license (http://creativecommons.org/licenses/by/4.0/).
